# Using a Developmental-Ecological Approach to Understand the Relation Between Language and Music

**DOI:** 10.3389/fpsyg.2022.762018

**Published:** 2022-02-18

**Authors:** Erica H. Wojcik, Daniel J. Lassman, Dominique T. Vuvan

**Affiliations:** Psychology Department, Skidmore College, Saratoga Springs, NY, United States

**Keywords:** development, language-music interaction, naturalistic observation, neurocognition, genetics, environmental input

## Abstract

Neurocognitive and genetic approaches have made progress in understanding language-music interaction in the adult brain. Although there is broad agreement that learning processes affect how we represent, comprehend, and produce language and music, there is little understanding of the content and dynamics of the early language-music environment in the first years of life. A developmental-ecological approach sees learning and development as fundamentally embedded in a child’s environment, and thus requires researchers to move outside of the lab to understand what children are seeing, hearing, and doing in their daily lives. In this paper, after first reviewing the limitations of traditional developmental approaches to understanding language-music interaction, we describe how a developmental-ecological approach can not only inform developmental theories of language-music learning, but also address challenges inherent to neurocognitive and genetic approaches. We then make suggestions for how researchers can best use the developmental-ecological approach to understand the similarities, differences, and co-occurrences in early music and language input.

## Introduction

In the last decades, scientists have made significant progress in understanding language-music interactions using an array of neurocognitive and genetic approaches. Despite the tacit assumption in this work that the environment affects the structure of neural substrates and the expression of genes, details of early language-music input have been comparatively understudied. The developmental-ecological approach to investigating the dynamics of early language-music input is an important complement to neurocognitive and genetic approaches that can make headway on some of the challenges language-music research has faced.

The developmental-ecological approach (Bronfenbrenner, 1979; West and King, 1987; Adolph, 2019) builds on the view that an adult’s brain and behavior is the product of interactions between the environment and biology throughout development. From this perspective, how we interact with our environment over time *is* what creates adult cognition, and so we must understand the conditions under which cognitive structures develop, from as early as possible, in as much detail as possible. Because this approach is rooted in the fact that development happens minute-to-minute, day-to-day, in a particular environment, it often involves the capture and description of continuous multi-modal recordings of infants’ daily life. Inherent to this approach is the acknowledgement that inter- and intra-cultural differences can have strong influences on developmental trajectories. For example, the developmental-ecological approach underlies Adolph’s work on motor development (Adolph et al., 2018), which pivoted the field away from describing motor behavior as the logical end of universal constraints and milestones, and toward an integrated perspective that can explain how different factors, from babywearing customs to nutrition, interact with the changing body across the lifespan. Like motor behavior, the processing of language and music should be seen as the result of culturally-dependent experiences that are constrained by social and physical affordances.

In this paper, we describe how understanding the biological and functional relation between language and music requires this developmental-ecological approach. First, we review the limitations of non-ecological approaches to studying language-music development. Then, we explain how a developmental-ecological perspective can address challenges inherent to neurocognitive and genetic approaches to language-music processing. Last, we make recommendations for conducting naturalistic research on early language-music input from a developmental-ecological perspective.

## Limitations of Non-Ecological Approaches to Investigating Language-Music Development

There is evidence within both music and language research that early experience affects later processing and behavior. Children’s early music environment, measured *via* parent report, is correlated with musical processing and musical success (Brand, 1986; Putkinen et al., 2013; Williams et al., 2015), and the quality and quantity of language exposure affects language processing (Grüter et al., 2014). Researchers have also investigated the interaction of music and language across development. Much of this work uses measures of experience or ability, such as years of music training or productive vocabulary at a certain age, to predict later brain activity and behavior. For example, some training studies have found that listening and learning how to play a musical instrument changes brain and behavioral responses to not only music stimuli, but language tasks as well (see Gordon et al., 2015, for review). Thus, early input, broadly defined, has been shown to affect how we respond to music and language, with some evidence of transfer across domains. This body of evidence has motivated domain-general, learning-based explanations for shared language-music processing (e.g., statistical learning theories such as Schön et al., 2008; Thiessen and Saffran, 2009, and Politimou et al., 2021; stimuli complexity theories such as Lebedeva and Kuhl, 2010; and familiarity-based theories such as Plantinga and Trehub, 2014).

However, the studies above treat early music and language experience as static, monolithic variables rather than continuous, complex streams that change across time. Asking a parent whether they have musical instruments at home (as some surveys do) is informative, but it ignores that the effect of instruments at home depends on the hour-to-hour and minute-to-minute dynamics of how infants interact with those instruments, and how that interaction in turn affects future neural activity and behavior. In order to understand the role of development and the environment in shared language-music processing, researchers need to move beyond summary measures of the environment and embrace the complex dynamics of how music and language input unfold over time.

## The Developmental-Ecological Approach: Embracing the Dynamics of Development

The developmental-ecological approach argues that to understand the interaction between language and music processing in adults, researchers must not only study children’s musical and language abilities in lab tasks, but also observe how children use the input to adapt to their environment in real-time. Thus, developmental-ecological studies: (1) record from naturalistic settings because adults and children interact differently in structured tasks vs. their daily routines (Tamis-LeMonda et al., 2017); (2) sample across many families, both intra- and interculturally, because childrens’ environments are inherently idiosyncratic and culture-dependent (Casillas et al., 2020; Montag, 2020); and (3) sample frequently and longitudinally because input interacts with development, such that what children see, hear, and touch differs from moment-to-moment and month-to-month (Kretch et al., 2014).

By describing how children engage with music and language across development—in the functional, social, and emotional context of their daily lives—we can develop models that predict language/music processing and production from actual measurements of the variability in early experiences and trajectories. This empirically-grounded approach differs from current models that often make assumptions about universal or cultural biases and experiences that unify or differentiate language and music. For example, studies on musical pitch structure often predict participant responses based on stimulus profiles summarized from musical corpora (e.g., Billboard Top 100, Bach Chorales), which serve as a proxy for musical experience (see for example the use of rock and classical corpora in Vuvan and Hughes, 2021). Developmental-ecological studies of the early music environment would allow researchers to discard such proxies and make predictions based on actual listener experience.

Some areas of language learning research have productively embraced the developmental-ecological approach. For example, in the 1970s, Brown (1973) used a combination of diary studies and tape recordings to describe early syntax and semantic development. The CHILDES database (MacWhinney and Snow, 1990) is a corpus of transcribed and tagged speech to children in both lab and naturalistic settings that has been used to create models of child language learning (e.g., Mintz, 2003). More recently, there has been a renewed interest in collecting day-long, longitudinal audiovisual recordings to describe the nuances of language input in first years of life (e.g., Roy et al., 2015; Tamis-LeMonda et al., 2019; Bulgarelli and Bergelson, 2020; Casillas et al., 2020; Sullivan et al., 2021). This work has led to novel findings, such as the role of context (Roy et al., 2015) and temporal structure (Casillas et al., 2020) in language development.

In contrast, scientific efforts to understand the early music environment largely do not stem from a developmental-ecological perspective, in part because this work has approached music input as an extracurricular, optional enrichment activity (e.g., Schellenberg, 2005). Researchers rarely record music in the daily lives of babies, but instead assess beliefs about and frequency of music engagement through surveys (Brand, 1985, 1986; Dai and Schader, 2002; Custodero et al., 2003; Custodero and Johnson-Green, 2008; de Vries, 2009; Valerio et al., 2012; Mehr, 2014; Williams et al., 2015; Politimou et al., 2021; Steinberg et al., 2021), individual and group interviews (Custodero, 2006; Young, 2008; Barrett, 2009, 2011; de Vries, 2009; Cho, 2015), and diary studies (Custodero, 2006; Barrett, 2009, 2011).

In the last two decades, some music researchers have begun to take an interest in direct observational research of the musical environment, through live observations (Barrett, 2009, 2011), lab recordings (Falk and Kello, 2017), and field recordings (Custodero, 2006; Barrett, 2009, 2011). This body of work has revealed that parents do value music in interactions with their children (Dai and Schader, 2002; Mehr, 2014; Cho, 2015), that music is used in routines, for social bonding, and in identity formation (Barrett, 2009, 2011), and that infants and children encounter music from a wide variety of sources (e.g., toys, singing, and recordings; Young, 2008). Recently, day-long at-home recordings have shown that infants are frequently exposed to live and recorded music, that the distribution of sources of music input is not uniform, and that critical features of music input impact singing development (Costa-Giomi and Benetti, 2020; Mendoza and Fausey, 2021a). Thus, there is increasing evidence that the developmental-ecological approach will lead to novel insights into early music processing.

Understanding language-music *interactions* will also benefit from the developmental-ecological approach. Current theories about why language and music have similar properties or why they activate the same neural substrates often assume that they share contextual and/or functional roles in human interactions. However, we have little data to empirically test this assumption, particularly in infancy and early childhood when neural specialization develops. For example, a prevalent theory is that both language and music evolved in part to facilitate social bonding (Savage et al., 2021). To support this theory, though, we need data that describes the social contexts in which babies hear language and music in their everyday lives, how babies respond to these stimuli, and how contexts and responses change over time. By describing the similarities and differences in how language and music are experienced in early development, researchers will also be able to form bottom-up hypotheses about cues that infants use to differentiate language and music (Brandt et al., 2012). Thus, a developmental-ecological approach will not only inform our understanding of language and music separately, but also how these domains overlap, separate, and interact across the lifespan.

## How Developmental-Ecological Research can Strengthen Other Approaches to Understanding Language-Music Interaction

The developmental-ecological approach will also address challenges faced by researchers investigating these questions from non-developmental perspectives. Below, we describe several prominent roadblocks to understanding language-music interaction and describe how a developmental-ecological approach could address them.

### Interpreting Correlations in Brain and Behavioral Measures

Cognitive neuroscientific studies comparing brain activity during language and music tasks generally find significant similarities across domains (for a recent review, see Schön and Morillon, 2019). For example, language and music syntax processing are co-located (e.g., in the bilateral inferior frontal cortex) and evoke similar neurophysiological responses (e.g., P600). However, these correlational data do not allow for the conclusion of shared processing of language and music in part because it is impossible to know whether these observed similarities constitute identical, homologous, or analogous neural signatures across domains.

By describing how neural responses to language and music change in response to learning across development, researchers can form more precise models about shared processing across domains. A developmental-ecological approach might measure an individual’s frequency of exposure to specific stimuli in day-to-day life and correlate these statistics to changes in neural markers like the activation in the inferior frontal cortex or the P600. This approach would be particularly powerful in a longitudinal context, allowing researchers to track the reciprocal shifts between changing stimulus frequencies and neural encoding of these patterns over the course of development.

### Designing Matched Language-Music Stimuli

The developmental-ecological approach can help researchers make better informed choices about stimulus design. For example, to investigate whether the processing of music syntax interferes with the processing of language syntax (and thus share resources), Fedorenko et al. (2009) manipulated sung materials in terms of “linguistic complexity” by comparing subject- and object-extracted relative clauses, and “musical complexity” by comparing in- vs. out-of-key notes. This design relies on an assumption that the operational definitions for linguistic and musical complexity, and the resulting choices of stimuli, are analogous.

These types of assumptions can be tested empirically by the developmental-ecological approach. In particular, understanding the distributions of language-music structures in early input would help theoretically motivate methodological decisions regarding stimulus selection and task design. For example, knowing the frequency with which individuals are exposed to subject- vs. object-extracted relative clauses, in- vs. out-of-key notes, and their co-occurrence across development would help with choosing analogous stimuli across domains. Moreover, descriptions of the variability in the quality of language and music input will help researchers decide if and how certain experimental stimuli are appropriate for use between individuals and across the lifespan.

### Interpreting Associations Between Language and Music Skills

Training paradigms test language skills before and after musical training in order to establish causal relations between music and language abilities (e.g., Douglas and Willatts, 1994; Forgeard et al., 2008; Schön et al., 2008; Bolduc, 2009; Degé and Schwarzer, 2011; Moreno et al., 2011; Bolduc and Lefebvre, 2012; François et al., 2013). These studies often do not fully consider pre-existing differences between groups, and these differences complicate the path between musical training and improved language processing (Swaminathan and Schellenberg, 2020; Vuvan et al., 2020; Wesseldijk et al., 2021). One way to develop clearer interpretations of training studies is to understand the developmental-ecological pathway of language and music learning in early life. Because training studies often start at school-age, understanding the variability in music and language input before that time point can help researchers better match participants across experimental conditions. More broadly, training studies implicitly assume that some music and language abilities only begin to develop when formal training starts. By describing language and music experience in the earliest years of life, researchers can measure the naturalistic “training” of language and music in infants and children, and thus understand the full ontological pathway both within and between the domains.

### Defining Phenotypes in Genetic Approaches

One approach to investigating language-music interaction is to locate genes that correlate with both language and music abilities. All genetic approaches to understanding language-music interactions, from twin and family aggregation studies (e.g., Peretz et al., 2007; Wesseldijk et al., 2021) to genome-wide association studies (GWAS; e.g., Reader et al., 2014; Niarchou et al., 2021), rely on researchers defining the phenotype to explore, which is fraught with difficulty. For example, the criteria that researchers use to define Specific Language Impairment affect the strength of heritability (Bishop and Hayiou-Thomas, 2008). Phenotype definition in language-music research will benefit from the developmental-ecological approach through empirical description of variability in language and music behavior in everyday life. The utility of the bottom-up creation of phenotypes can be seen in bilingualism research, where descriptions of early bilingual environments have led to a more nuanced understanding of how to define, measure, and study bilingualism and its cognitive outcomes (Byers-Heinlein et al., 2020). By describing the early language-music environment, we can develop a more valid spectrum of language-music phenotypes to use in genetic research.

## Recommendations for Conducting Developmental-Ecological Research on the Early Language-Music Environment

In the past two decades, there has been a surge in naturalistic work in developmental psychology (Yoshida and Smith, 2008; Adolph, 2016; Bulgarelli and Bergelson, 2020; Sullivan et al., 2021) which has yielded several insights into the design and interpretation of naturalistic data. For example, researchers have shown that first-person view head cameras provide a more valid recording of what infants are visually attending to in their environment than traditional third-person recordings (Yurovsky et al., 2013), and others have examined assumptions embedded in hour-long vs. full day recordings (Bergelson et al., 2019; see Mendoza and Fausey, 2021b, for an introduction to thinking through decisions surrounding who, what, where, and how to build and code large, naturalistic corpora). Here, we give recommendations specific to studying the early language and music environment in tandem, from a developmental-ecological perspective.

### Identify Theoretical Assumptions Behind Coding Schemes

In naturalistic work, researchers need to explicitly define coding schemes. For example, observational research of children’s music-making requires the *a priori* creation of coding schemes that explicitly state what “music” and “music-making” are. When coding for bouts of music, researchers could define music by its rhythm, pitch, context of production, etc. These definitions have different theoretical assumptions about what infants perceive as music input (vs. language or environmental noise). For example, a parent chanting “Come here, baby” in infant-directed speech might be coded as music because of its pitch and rhythm, but not in a context-of-production framework. These three definitions of music are all valid, but the resulting data from choosing one over the other must be interpreted within the limitations and assumptions of the underlying theory in mind.

In fact, one benefit of naturalistic work is that it surfaces previously unattended theoretical assumptions that impact research design and analyses. For example, a survey item such as “My child rarely makes music” (Politimou et al., 2018) relies on the parents’ lay definition of music and music-making. This item inherently assumes that the parents’ definition of music is the same as the child’s and the researchers’. Thus, naturalistic work allows for the development of evidence-based, bottom-up definitions of language and music that potentially evolve across the lifespan.

### Study Language and Music as Both Analogous and Distinct

In naturalistic work, researchers should conceive of music and language as both analogous and distinct domains. An *analogous approach* asks the same question in both domains to examine similarities and differences. Under this approach, researchers port theoretical and experimental frameworks from one domain to the other. For example, we know that most North American infants receive the majority of their language input from a single source (the primary caregiver; Sperry et al., 2019). One might then run the analogous analysis in the music domain by measuring the proportion of music input from the primary caregiver vs. other people, and finally compare the language and music source distributions.

Although taking an analogous approach can be informative, it can obscure functional differences between the two domains. A *distinctive approach* considers each domain separately, enabling researchers to discover, from the bottom up, phenomena and mechanisms that are unique to language or music. Observational naturalistic research (from the developmental-ecological approach) provides the opportunity to study each domain in its own context. For example, the use and quality of recorded music in North American households (from baby toys, streaming services, etc.; see Mendoza and Fausey, 2021a) is distinct from the use and quality of recorded language (from radio, podcasts, etc.), and this domain difference likely changes across development. Accordingly, theories of how infants learn from live vs. recorded sources may differ between language and music, and these theoretical differences may lead to distinct research approaches in each domain.

The usefulness of studying language and music input as both analogous and distinct can be seen in psychological research on signed and spoken languages. Some research focuses on analogies between signed and spoken languages (Petitto et al., 2000), whereas other work describes how functional differences between signing and speaking underlie unique features of both (Senghas, 2010). This has led to a nuanced understanding of the neurocognitive mechanisms that subserve languages in different modalities.

Using analogous and distinctive frameworks in tandem can also address the historical tendency to study the language and music of predominantly white, WEIRD communities (Schwab and Lew-Williams, 2016; Baker et al., 2020; Jacoby et al., 2020). This tendency has resulted in researchers treating white, WEIRD communities as the human default, and over-generalizing data obtained from this population. In the same way that analogous and distinctive frameworks are useful in investigating language-music interactions *within* a particular cultural context, this dual framework is also useful in investigating language-music interactions *between* cultural contexts. An analogous approach might lead researchers to replicate past work in non-white, non-WEIRD populations, whereas a distinctive approach would start by observing and describing the variability in music and language input in different cultures.

### Collaborate

In naturalistic work, researchers should collaborate in order to combine methodological and theoretical expertise as well as to share logistical resources such as datasets, analysis scripts, coding schemes, training materials, and more. Generous collaboration will give more researchers the ability to engage with the developmental-ecological approach. Language and music studies each exist at a nexus of interdisciplinary contact between behavioral scientists, humanities scholars, and artists. Scholars trained in different disciplines will thus bring contrasting values, theories, and methods to questions that bridge these two domains. For scientists whose training is predominantly experimental, the developmental-ecological approach offers an exciting opportunity to leverage the methodological expertise of colleagues trained in qualitative and observational research. For example, there is a rich history of qualitative and quantitative observation within musical corpus studies (Shanahan, in press) and linguistics (Brown and Levinson, 2018). Experimentalists should build on the theory and logistical insights from this past work and launch new interdisciplinary collaborations. Researchers should also embrace opportunities to investigate language-music interactions *via* secondary analysis of existing naturalistic developmental datasets (e.g., Cirelli et al., 2020). There are several platforms for ethically sharing audio/video datasets and analysis tools (e.g., Databrary; Gilmore et al., 2015) and active communities for further resource sharing (e.g., DARCLE, 2022).

## Conclusion

To fully understand the gene-brain-environment dynamics that give rise to adult cognition, we must have a clearer understanding of the dynamics of the environment in early development. Language-music researchers universally acknowledge that children learn about language and music implicitly, but tend to skirt important questions about how this learning unfolds over time and how the learning process influences downstream processing throughout the lifespan. The developmental-ecological approach offers a path for specifying developmental trajectories and mechanisms that will in turn advance theories of language-music interaction.

## Author Contributions

EW and DV contributed equally to the article and conceived of the argumentative structure. EW, DL, and DV wrote the text. All authors contributed to the article and approved the submitted version.

## Funding

This research was supported in part by a grant from the Grammy Museum Foundation.

## Conflict of Interest

The authors declare that the research was conducted in the absence of any commercial or financial relationships that could be construed as a potential conflict of interest.

## Publisher’s Note

All claims expressed in this article are solely those of the authors and do not necessarily represent those of their affiliated organizations, or those of the publisher, the editors and the reviewers. Any product that may be evaluated in this article, or claim that may be made by its manufacturer, is not guaranteed or endorsed by the publisher.
